# Analysing the potential impacts of three interventions on fruit and vegetable consumption in urban Kenya using participatory systems modelling

**DOI:** 10.1017/S1368980023002033

**Published:** 2023-12

**Authors:** Charles F Nicholson, Eva Monterrosa

**Affiliations:** 1 Department of Agricultural and Applied Economics, University of Wisconsin—Madison, 442 Animal Science Building, 1675 Observatory Drive, Madison, WI 53706, USA; 2 Global Alliance for Improved Nutrition, Geneva, Switzerland

**Keywords:** Nutrition, Group Model Building, Systems modelling, Fruit and vegetable consumption, Kenya

## Abstract

**Objective:**

This study uses participatory modelling with stakeholders to assess the potential impacts of three interventions intended to increase fruit and vegetable (F&V) consumption in urban Kenya.

**Design:**

A participatory process using Group Model Building (GMB) developed a conceptual model of the determinants of vegetable consumption. A subsequent quantitative System Dynamics model using data from primary and secondary sources simulated vegetable consumption from 2020 to 2024 under three proposed interventions suggested by stakeholders: increasing consumer awareness, reducing post-harvest losses and increasing farm yields. Model analyses assumed mean parameter values and assessed uncertainty using 200 simulations with randomised parameter values.

**Setting:**

The research was implemented in Nairobi, Kenya with simulation analyses of mean per capita consumption in this location.

**Participants:**

Workshops convened diverse F&V value chain stakeholders (farmers, government officials, NGO staff and technical experts) to develop the conceptual model, data inputs and intervention scenarios.

**Results:**

Increasing consumer awareness was simulated to increase vegetable consumption by relatively modest amounts by 2024 (5 g/person/d from a base of 131 g/person/d) under mean assumed value of value chain response parameters. Reducing perishability was simulated to reduce consumption due to the higher costs required to reduce losses. Increasing farm yields was simulated to have the largest impact on consumption at assumed parameter values (about 40 g/person/d) but would have a negative impact on farm profits, which could undermine efforts to implement this intervention.

**Conclusions:**

The combination of GMB and simulation modelling informed intervention priorities for an important public health nutrition issue.

Fruit and vegetable (F&V) consumption is considered an important component of a healthy diet with numerous documented health benefits^(1,2)^. In urban Kenya, a high proportion of the population consumes F&V, but the quantities are below the amount recommended by the WHO of 400 g/d for all socio-economic groups reported in the Global Dietary Database^(3)^ (Fig. 1). Consumption of both fruits and vegetables in Kenya is also considerably below the ‘optimal’ levels proposed of 300 and 400 g/d^(2)^, respectively. Moreover, there has been essentially no change in per capita consumption of fruits and vegetables on average in Kenya during the years 2000 to 2015 based on data from the Global Dietary Database, despite a threefold increase in GDP per capita during this period^(4)^.

**Fig. 1:**
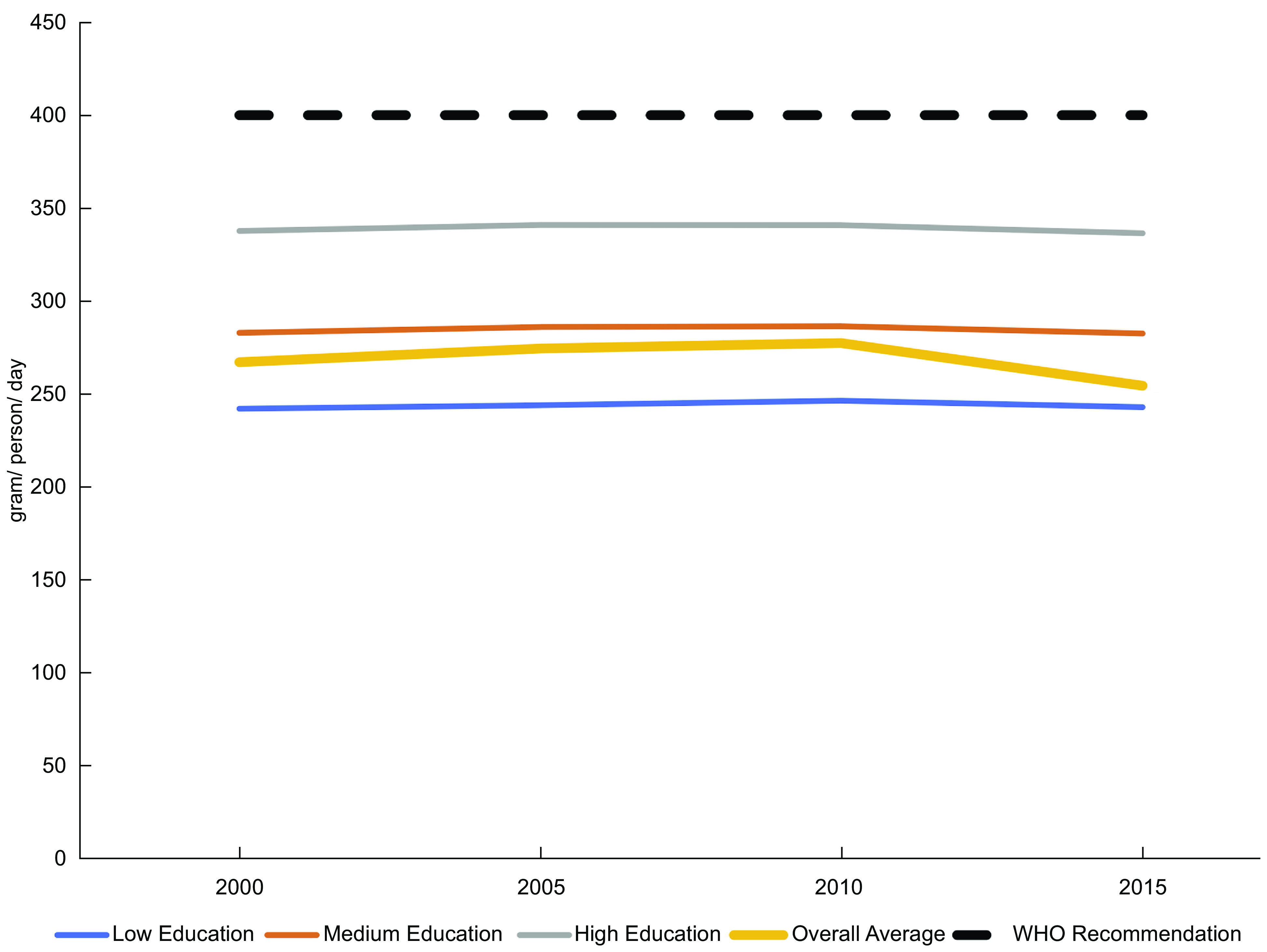
Reference mode: per capita consumption of fruits and vegetables, daily average by educational attainment, Kenya, 2000 to 2015. Note: Global Dietary Database data are now available for 2020 but were not at the time of the first GMB workshop so they are not shown here. Educational attainment is a proxy for socio-economic status

Low and stagnant levels of F&V consumption in Kenya during 2000–2015 provide the motivation for an *ex ante* evaluation of interventions that could increase consumption to WHO recommended levels. Previous literature has identified diverse factors likely to affect F&V consumption both more generally and specifically in the Kenyan context^(5,6)^. These factors typically fall into one of three general categories. *Availability* comprises production and post-production supply chain activities that facilitate product purchases by ultimate consumers. *Affordability* means that regular purchases of product are possible by consumers based on their incomes and product prices but could also include the time costs required for purchases. *Desirability* comprises a diverse set of influences that affect a consumer’s willingness to purchase F&V, including cultural influences, knowledge of both benefits and preparation, product quality, safety and hygiene and emotional responses to these food choices.

Although numerous factors have been identified as influencing consumption of fruits and vegetables in Kenya^(5–9)^, this information alone is generally not sufficient to assess interventions to increase F&V consumption. Much of the available information about consumption determinants is qualitative and does not allow a quantitative assessment of the relationship between determinants and consumption levels. Another is that even when determinants are better understood (quantitatively) the development of effective interventions does not always follow directly from this information, given a multiplicity of possible intervention approaches designed to influence the determinants. Finally, interactions among decision-makers throughout the F&V supply chain have the potential to enhance or limit the effectiveness of interventions to increase consumption and these have not been analysed in previous work.

The current state of knowledge thus constrains the identification and implementation of priority interventions to increase F&V consumption in urban Kenya. To the best of our knowledge, there has been no systematic comparative evaluation of the wide range of intervention possibilities in this context. Thus, an initial comparative evaluation of interventions would be useful to support priority setting for organisations with a mandate to support healthy eating, for example, through increasing F&V consumption, and is the overarching motivation for this study. To account for the multiple interacting elements of the value chain that could influence the effectiveness of interventions, we apply a systems framework^(10)^ combined with stakeholder engagement in the development and quantification of an analytical model.

The overall objective of this study is to evaluate interventions to increase F&V consumption in urban Kenya, with the following specific sub-objectives:

1. Identify hypothesised causal pathways that result in lower-than-desired F&V consumption and potential interventions to increase that consumption;
2. Develop a quantitative model to represent the interacting factors that constrain F&V consumption and facilitate *ex ante* assessment of proposed interventions; and
3. Assess the effectiveness of three interventions commonly proposed by international organisations: increasing consumer awareness of the health benefits, reducing perishability in the value chain and increasing farm yield (production).

## Methods

### Group Model Building process with fruit and vegetable supply chain stakeholders

This study combines information from the Group Model Building (GMB) approach^(11,12)^ with a review of information from the literature to assess intervention options, with a focus on how they would increase average F&V consumption during the 5 years following assumed implementation^(13)^. GMB processes have previously been applied in public health nutrition^(14–16)^ but primarily to develop qualitative conceptual models with stakeholders. Relatively few studies^(17)^ have evaluated public health nutrition programmes using quantitative systems modelling approaches.

We convened two GMB workshops, the first in September 2019 to develop an initial conceptual model and identify potential interventions. A second workshop was held with the same participants in April 2020 (via Zoom due to Covid restrictions) to present the structure of the quantitative System Dynamics model to stakeholders, to solicit suggested modifications and to determine priority interventions for quantitative analysis. The first GMB workshop solicited input from relevant stakeholders about the factors and linkages that result in persistent insufficient F&V consumption in Nairobi. An additional objective was to increase awareness of the complexity of supply chain interactions that could affect the impacts of interventions designed to increase F&V consumption. This workshop was held in Nairobi as two half-day sessions on 12–13 September 2019 and included sixteen participants in different roles (Table 1) in the Kenya supply chain for F&V. These stakeholders were selected through contacts of the sponsoring organisation, which gave greater emphasis to behavioural change approaches to increasing vegetable consumption. Consistent with the GMB process, participants were led through a series of activities based on GMB scripts, for example, ‘Initiating and Elaborating a Causal Loop Diagram or Stock and Flow Model’^(18)^ to identify factors causing the low and stagnant levels of F&V consumption as the reference mode behaviour through 2025. During the introduction, participants were provided with information about the overall process for the project, the structure of the workshop, an operational definition for fruits and vegetables and an illustrative listing of F&V supply chain stakeholders.

**Table 1:** Workshop participants

Role in vegetable value chain	Number of participants	Organisations
Production	2	Farm operator Ministry of Agriculture
Distribution	1	Private company
Technical support	2	Consulting companies for value chains development
Research	3	University researcher, behaviour change communication organisation, international agricultural research organisation
NGO	8	Representatives of local and international NGO with interest in agriculture and in improved diets

**Fig. 2:**
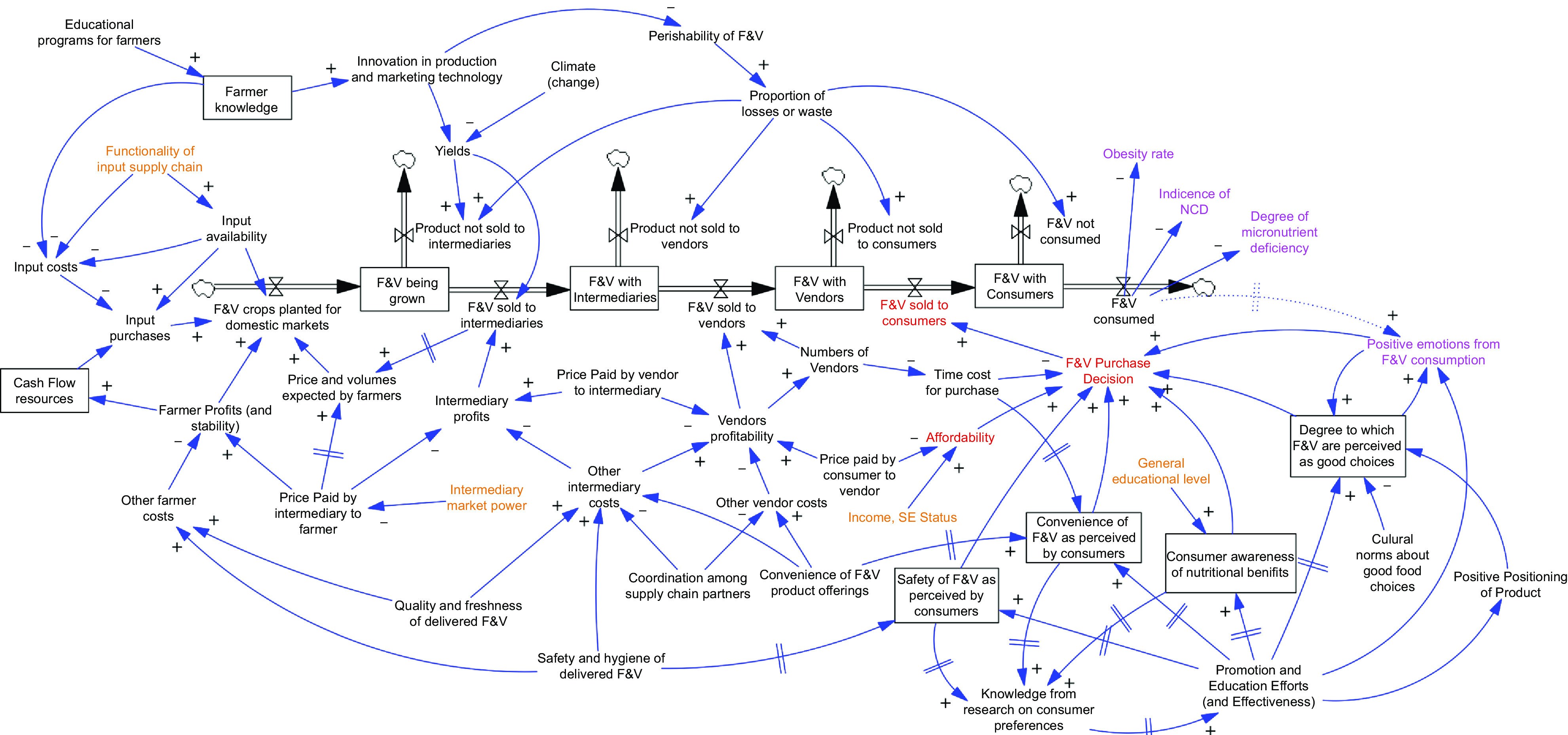
Initial systems mapping based on day 1 exercises from September 2019 stakeholder workshop. Note: Arrows represent hypothesised causal relationships, where + indicates that a change in the initial variable results in a change in the same direction as the variable to which the arrow points. Red variables are key outcomes, orange exogenous factors and pink ultimate health outcomes of interest

The second half-day session began with a summary of the factors that participants identified as influencing consumption of fruits and vegetables in Nairobi and their definitions and metrics for affordability, availability and desirability. The initial systems diagram based on stakeholder input was discussed to identify any necessary corrections or additions (Fig. 2). A number of feedback processes with the potential to enhance or limit increases in F&V consumption were identified qualitatively to illustrate the potential insights from a systems analysis. Participants then identified priority interventions to increase F&V consumption, that is, to modify the ‘reference mode’ behaviour (i.e. per capita consumption in g/d) to more desirable outcomes.

### Quantitative model development

Based on the input from stakeholders from the first workshop, a quantitative simulation model based on the System Dynamics approach was developed for quantitative assessment of proposed interventions. The structure for the supply chain components of the model (farm production, intermediaries and vendors) is based on a previous System Dynamics supply chain formulation^(19)^ modified in this case to reflect multiple linked supply chain actors for F&V products. Intermediaries are defined for the purposes of the model as the first buyer of product from farmers and the sellers of product to vendors, who are assumed to sell directly to individual consumers (households). This is a simplification in the sense that there can be multiple intermediaries between farmers and vendors, but this aggregation likely does not affect the outcomes of the model.

Prices from sellers to buyers are determined by inventory coverage (the amount of product in storage at a market level divided by current sales and expected product losses – spoilage). Sales prices generate revenues, which along with costs for production and distribution determine profits. Profitability of farmers, intermediaries and vendors determines the level of initiation of new production (for farms) or marketing (purchases/orders, for intermediaries and vendors), which become part of available inventories with a delay (e.g. time is required to increase production and to contract for purchases and receive deliveries from suppliers). Prices also determine the demand for product by intermediaries, vendors and consumers.

Although in some supply chain models, perfect coordination is assumed (orders are coordinated throughout all levels of the supply chain), we do not assume that the F&V supply chain for Nairobi demonstrates this degree of coordination. Rather, farmers, intermediaries and vendors are assumed to operate independently and thus may make supply or purchase decisions not entirely aligned with the purchase or production decisions of supply chain partners. Potential intervention points are represented for each of the market actors. Relevant literature on F&V supply chains in Kenya^(7,20–23)^ and related to consumer behaviour^(1,2,5,6,8,9,24,25)^ was used to develop specific quantitative relationships among the variables identified in the stakeholder workshop.

The initial model is designed to replicate the reference mode of observed limited growth in F&V consumption per capita. The current model version represents 2015 observed consumption levels in ‘dynamic equilibrium’ beginning in 2018 with unchanged market or promotion conditions and then examines the impacts of changes to factors that would affect consumption. The model represents 5 years (with a weekly time unit of observation) starting with 2018. The current model focuses only on a single ‘generic’ product that is more representative of leafy greens. A detailed model description is provided in supplementary materials.

### Specification of model scenarios for analysis of priority interventions

Intervention scenarios were developed with the stakeholders based on potential actions identified in the first workshop. Highest-priority interventions were identified with a ranking process during the second workshop, and initial input from stakeholders informed the possible scope of changes and impacts. When needed to define the scenarios, additional information was collected from individual stakeholders (e.g. from a farmer participant about the potential for yield increases and the associated costs). This process defined the potential impacts of interventions (Table 2) and scenarios (Table 3). These scenarios analysed interventions that focused on either consumers or other value chain participants (farmers or intermediaries). For this paper, we focus on three commonly proposed interventions: increasing awareness of the health benefits of F&V consumption, reducing perishability in the supply chain and increasing vegetable yields. Each of these has been attempted to some extent previously in the Kenya F&V supply chain and is otherwise commonly proposed interventions to improve nutritional outcomes from food supply chains more generally^(26,27)^. To facilitate comparison among scenarios, the assumptions about changes are typically expressed in terms of percentage changes from the current situation, for example, a 10 % increase in the proportion of the population that is aware of relevant nutritional benefits of F&V consumption. The ranges of possible percentage changes were based on input from the second workshop and subsequent discussions with individual subject matter experts.

**Table 2:** Summary of characteristics of priority potential interventions to increase fruit and vegetable consumption based on April 2020 workshop and subsequent consultation with subject-matter experts

Intervention characteristic	Improve awareness of nutritional benefits (consumer focus)	Reduce farm perishability (farm focus)	Increase yields (farm focus)
Measurable indicator	Number of servings, portion sizes, diversity (adding new fruits and vegetables, not just more of same)	Proportion of production harvested not suitable for sale	Production per acre, kg/acre
Degree of change possible	Varies with type of awareness, from limited to moderate, but limited information is available for specific actions	Could be reduced to 10 % (compared to currently assumed 15 %)	100 % increase
Actions required by supply chain actors or external partners	Programme efforts to increase awareness	Farmer training in Good Agricultural Practices (GAP); improved storage, continuous market access (especially in rains)	Farmer training in Good Agricultural Practices (GAP); increased investment and input use
Impact on supply chain costs	Limited direct impacts	Increases, varies with intervention	May reduce unit costs of production although total costs are higher
Time required to implement	Potentially lengthy	Potentially lengthy for farmer training and infrastructure development	Potentially lengthy for farmer training and infrastructure development
Other comment	Awareness of general nutritional benefits is already high, so awareness efforts would need to focus on other aspects. Stepped progress to meet goals may be appropriate strategy	Perishability can be linked to yields but is treated separately here	Yields can be related to perishability but are treated separately here

**Table 3:** Changes in simulation model parameters to implement intervention scenarios and related sensitivity analyses

Simulation model changes	Improve awareness of nutritional benefits (consumer focus)	Reduce farm perishability (farm focus)	Increase farm yields (farm focus)
Parameters modified for scenario	10 % increase in awareness No change in value chain costs	33 % reduction in post-harvest perishability at the farm level (10 % losses rather than 15 % losses) 10 % increase in unit variable costs of production at the farm level	50 % increase in yields at the farm level 5 % increase in unit variable costs of production at the farm level
Range of value for sensitivity analysis	None	5–20 % increase in unit variable costs of production at the farm level	5 % decrease to 10 % increase in unit variable costs of production at the farm level

Changes in relevant value chain costs associated with implementation of the intervention are also expressed in terms of percentage changes from the current values. All interventions are assumed to be implemented (and are fully effective) as of May 2020. This assumes no one-time costs (investments), time delays or issues with implementation, which is consistent with the focus of the model but represents a best-case scenario in terms of impacts vis-à-vis more realistic programme implementation challenges. Thus, these scenarios provide a high-level and best-case analysis that could be complemented by formal assessment of implementation activities.

### Sensitivity analysis of priority interventions with uncertainty in model inputs

The values of many parameters describing the response of supply chain actors are not well known, and the participants in the April workshop expressed different opinions about many of them. Given the uncertainty about these values, it is important to evaluate how alternative assumptions about them affect the impacts of the priority interventions. To do this, we specified likely ranges of values from many of the uncertain parameters and used these ranges to simulate 200 random combinations of uncertain parameters for each of the interventions and their combination. Lacking good information about the nature of the distribution for these parameter values, for all of them we assumed a uniform probability distribution for maximum and minimum values reviewed by stakeholders, using the ‘multivariate’ selection approach provided by Vensim™. The sensitivity analyses assumed the same expected magnitude of impact of interventions as in the deterministic scenarios (e.g. a 10 % increase in awareness) but simulated a range of outcomes based on distributions of cost changes and response parameters. These analyses provide a probability distribution of values for vegetable consumption specifically, which is appropriate given the uncertain nature of value chain behavioural responses.

The impact of alternative parameter values was assessed using a linear regression of the end-of-2024 consumption level on the parameter values used in the 200 simulations for the stochastic analysis. The sign and magnitude of these regression coefficients can be used with information about the low and high values of the parameters used in the 200 simulations to assess the impacts on consumption of changes in specific parameters.

## Results

### Causal Loop Diagram development and analysis

**Fig. 3:**
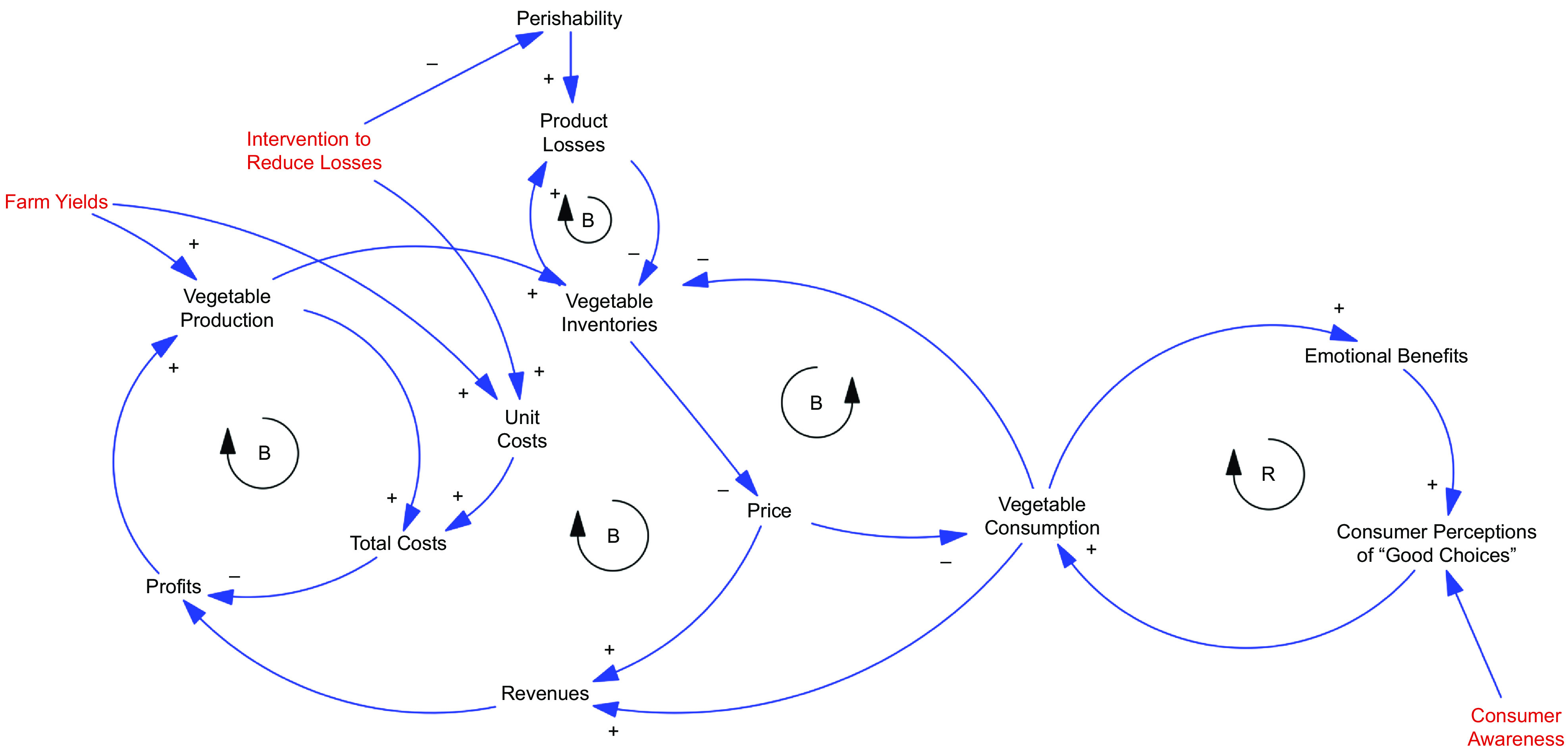
Casual loop diagram of key feedback effects for the three priority interventions. Note: Arrows represent hypothesised causal relationships, where ‘+’ indicates that a change in the initial variable results in a change in the same direction as the variable to which the arrow points and ‘-’ indicates that a change in the initial variable results in a change in the opposite direction as the variable to which the arrow points. Red text identifies intervention points selected as priorities by stakeholders. ‘B’ and ‘R’ denote the polarity of the loops, balancing (negative) and reinforcing (positive) feedback loops, respectively

As noted previously, an initial systems diagram (Fig. 2) based on stakeholder input was discussed at the first workshop to identify necessary corrections or additions. At the second workshop, this diagram was simplified and formulated as a Causal Loop Diagram to identify key feedback processes for the highest-priority interventions (Fig. 3). Conceptual analysis with this diagram indicated that an intervention to increase awareness could increase consumer perceptions of making good choices and the emotional benefits derived from them, with a positive feedback effect on consumption. An offsetting effect due to the impact of increased consumption on inventories and higher prices was also present. Reductions in perishability would initially increase costs, but initial lower product losses would also increase inventories and lower prices. The net effect of the intervention on consumption is difficult to determine from the diagrammatic analysis alone. Increasing farm yields would increase inventories, lower prices and support increased consumption, but lower prices could also reduce farm profits.

### Results of deterministic intervention scenarios

**Fig. 4:**
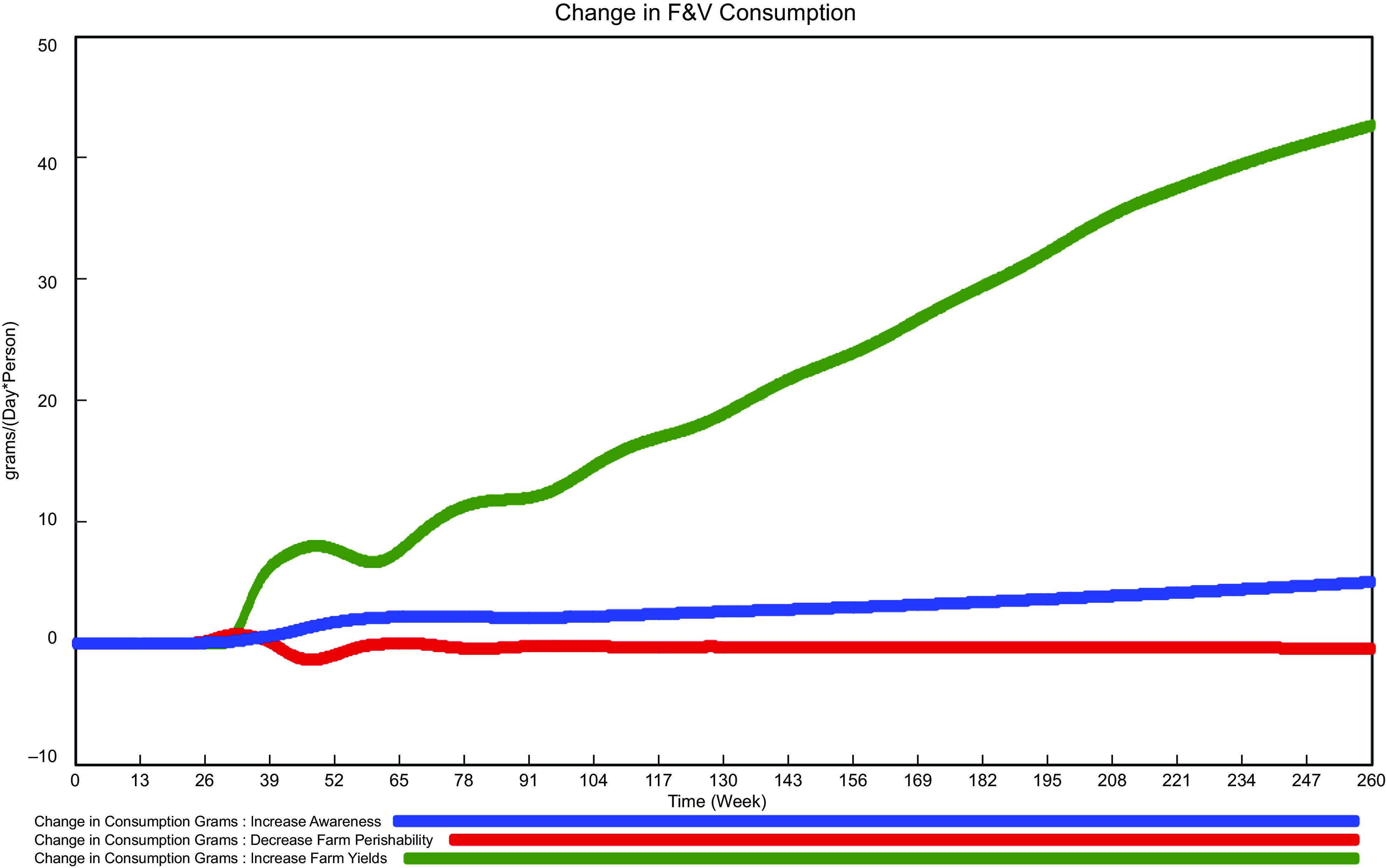
Simulated changes in daily per capita vegetable consumption under three intervention scenarios. Blue: increase consumer awareness. Red: reduce farm perishability. Green: increase farm yield

A first set of scenarios assessed the impacts of the three interventions at the mean estimated values of key response parameters and thus represented the mean expected impact of the interventions. They also provide a starting point for discussion of stochastic scenarios when the impacts of ranges in uncertain parameters are assessed. As discussed further below, alternative parameter assumptions will affect the degree to which any of the intervention can be effective, for which determining the distribution of values can be useful. The deterministic results indicate that increasing consumer awareness and increasing farm yields would increase vegetable consumption (Fig. 4; Table 4), the latter effect due to increased supply and therefore lower prices. These two interventions result in continuous increases during the 5 years simulated by the model. This pattern of ongoing increase results from the time required for supply chain actors to perceive and respond to relevant changes and from a reinforcing feedback effect. Although programmes are assumed to be implemented instantaneously and immediately effective, the behaviour of value chain participants is assumed to require time for changes to occur. This latter effect is based on the positive emotional response and reinforcement of vegetables as good choices, both of which are assumed to be enhanced as vegetable consumption increases. Thus, initial increases from the intervention are maintained and enhanced by the emotional response processes of consumers.

**Table 4:** Simulated impacts of interventions on vegetable consumption, prices received and profits for supply chain actors

Simulated outcome	Dynamic equilibrium	Increase awareness	Decrease farm perishability	Increase farm yields
Average daily per capita vegetable consumption, g/d	131·2	134·0	130·9	153·0
Ending daily per capita vegetable consumption, g/d	131·2	136·2	130·8	173·8
Average total quantity consumption, million kg/week	3·02	3·08	3·01	3·52
Ending total quantity consumption, million kg/week	3·02	3·13	3·01	4·00
Average price received, KSh/kg				
Farm	22·0	22·7	22·5	17·6
Intermediary	45·0	46·5	45·2	45·6
Vendor (paid by consumer)	53·7	55·4	53·7	56·0
Average total profits, million KSh/week				
Farm	31·7	34·6	29·0	16·3
Intermediary	23·5	26·7	22·2	48·3
Vendor	7·5	8·7	7·1	15·9

Another commonly proposed supply chain intervention is reduction of perishability^(20,22,23)^ often with the goal of reducing costs due to product losses and also increasing product availability. (Reducing losses can also have the goal of reducing environmental impacts, although that is not a focus herein.) Our scenario assumes a one-third reduction in losses of product from post-harvest farm inventories, from their currently estimated value of 15 % to a value of 10 %. To achieve this reduction in farm perishability, a 10 % increase in variable costs is assumed to be required, which reflects the potential costs of improved storage and farmer training. This intervention is simulated to reduce daily per capita consumption of vegetables.

The intervention with the largest impact on simulated vegetable consumption is for increased farm yields. By the end of 2024, this scenario suggests that daily per capita consumption could be increased by nearly one-third of 2020 amounts, by more than 40 g/person/d. This large change is due in part to the assumed size of the yield increase (50 %), the relatively small increase in unit costs of production to assume this yield increase (5 %) and the fact that the large increase in production from higher yields results in a 20 % reduction in the farm price.

### Results of stochastic intervention scenarios

**Fig. 5:**
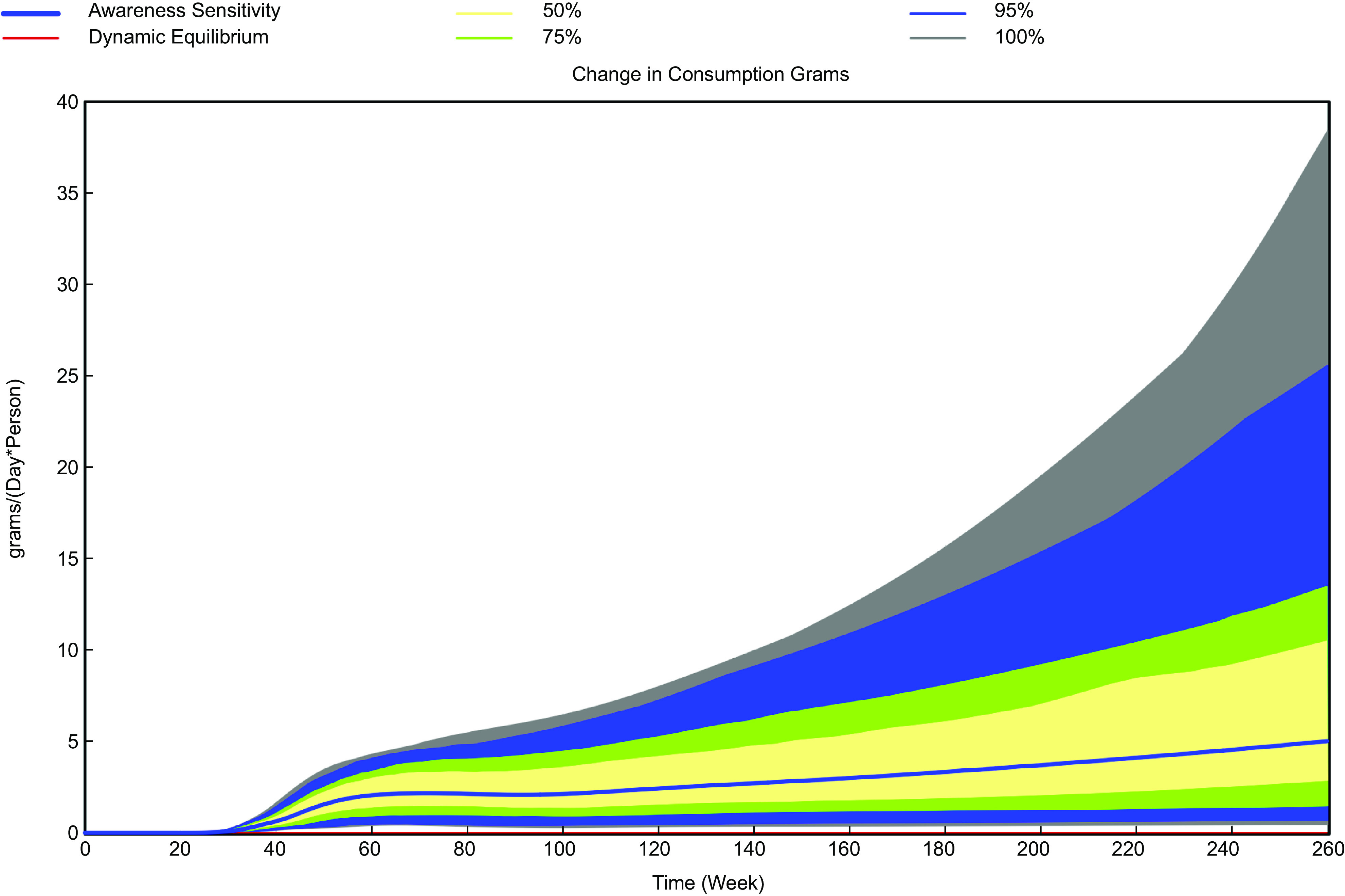
Simulated range of changes in daily per capita vegetable consumption with 10 % increase in awareness of nutritional benefits, *n* 200 random sets of parameter values

**Fig. 6:**
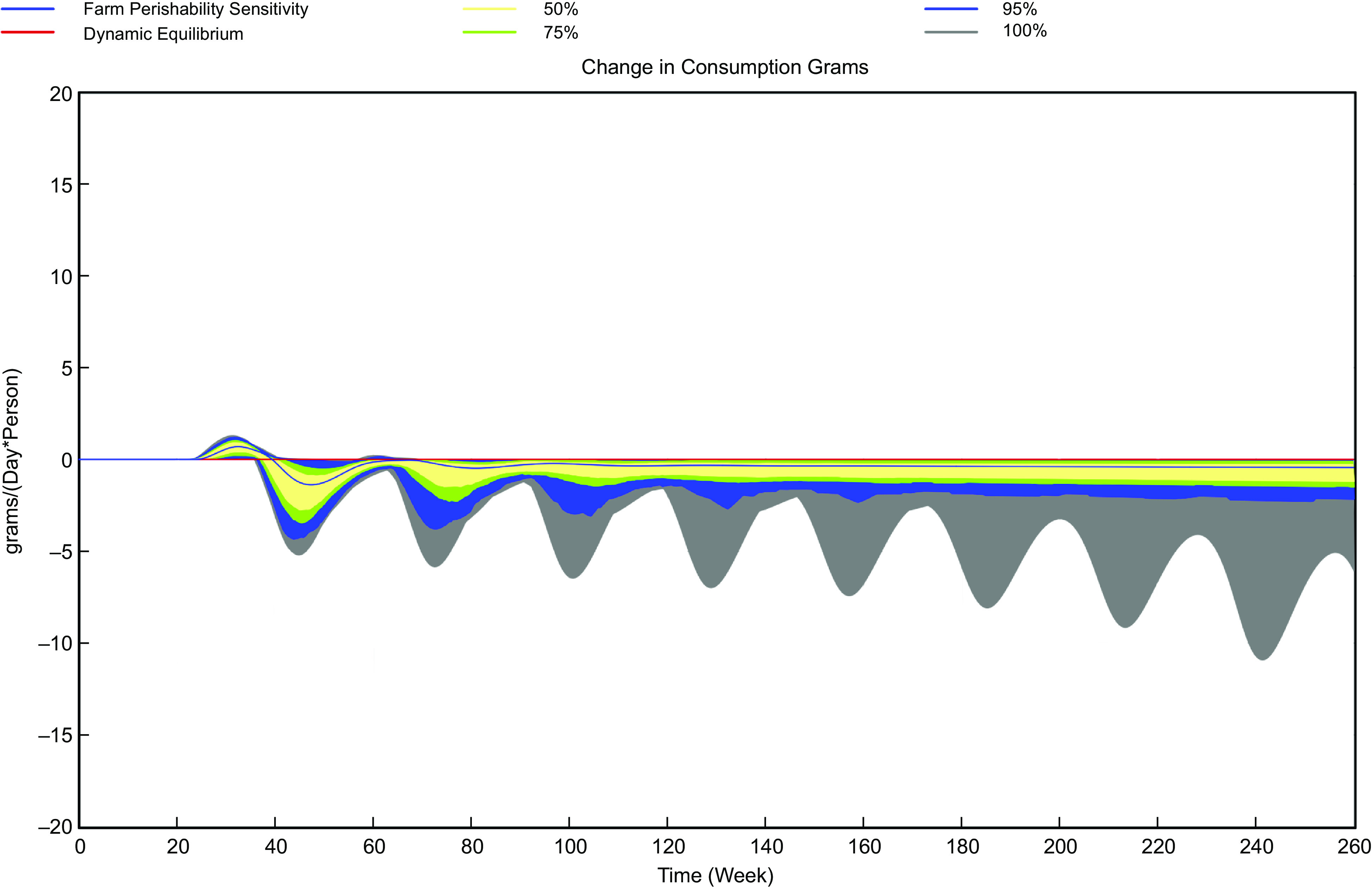
Simulated range of changes in daily per capita vegetable consumption with a 33 % reduction in farm perishability, *n* 200 random sets of parameter values

**Fig. 7:**
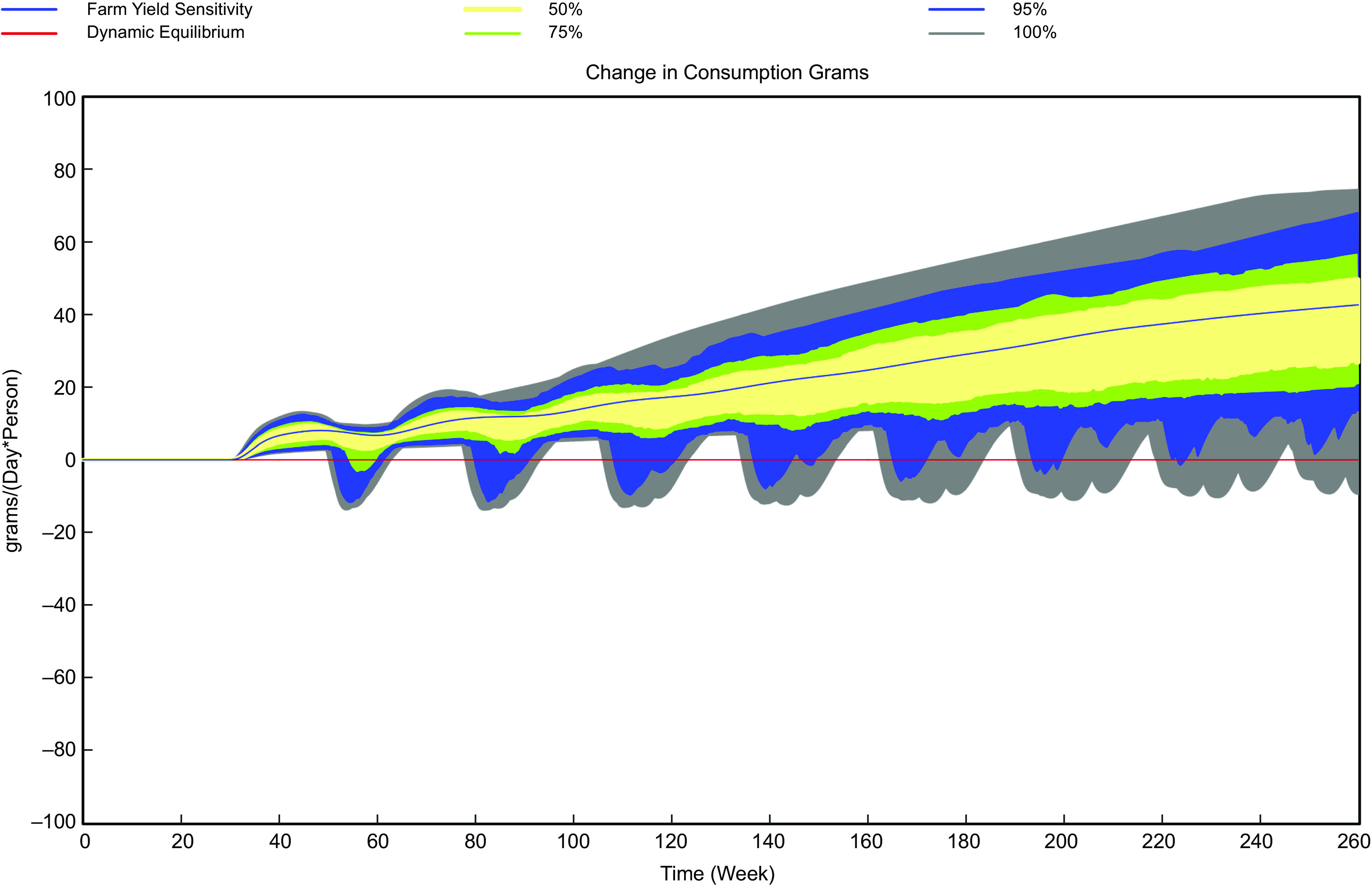
Simulated range of changes in daily per capita vegetable consumption with 50 % farm yield increase, *n* 200 random sets of parameter values

The stochastic analyses indicate that the range of impact of the three interventions on simulated vegetable consumption can vary based on assumptions about value chain responses and the effect on value chain costs (Table 5). The range of values at the end of 2024, which tend to be the highest values of consumption for reasons discussed previously, varies by only 6 g/person/d for scenario of reduced perishability but is larger of the scenarios modifying consumer awareness (nearly 40 g/person/d) and increasing farm yields (84 g/person/d) (Table 5 and Figs. 5–7). The range of consumption outcomes at the end of 2024 is greater than 10 % of current consumption for the scenarios except for the reduced perishability scenario, which suggests the importance of understanding which uncertain parameters have the largest impact on the outcomes.

**Table 5:** Range of simulated impacts of interventions on 2024 vegetable consumption, *n* 200 random sets of parameter values

Simulated outcome	Increase awareness	Decrease farm perishability	Increase farm yields
Average daily per capita vegetable consumption, g/d			
Minimum value	131·6	125·1	121·6
Median value	136·9	130·5	171·3
90^th^ percentile value	149·8	131·2	194·4
Maximum value	169·6	131·2	205·6
Range of values (maximum – minimum)	38·0	6·1	84·0

Note: Consumption in dynamic equilibrium is 131·2 g/person/d.

*Source: Authors’ calculations.

The regression analysis indicates the impact of stochastic factors on ending F&V consumption. As an example, for the scenario increasing awareness of nutritional benefits, a 10 % increase in awareness is assumed, but the sensitivity of consumption to awareness is uncertain, with a range of values from 0·2 to 1·0. Given the regression coefficient for sensitivity of consumption to awareness, the impact of a change from 0·2 to 1·0 in the value of the sensitivity indicates that the impact of changing from the low and high values of sensitivity is about 16 g/person/d. A value of 16 g/person/d is about 40 % of the observed range of values, suggesting that uncertainty in this parameter accounts for a substantive amount of the 38 g/person/d uncertainty observed for the range for this intervention assuming other factors held constant. For the perishability scenario, the only important parameter was the assumed cost, which accounted for two-thirds of the variation in 2024 outcomes. For farm yields, assumptions about the cost changes required for yield (a 5 % decrease to a 10 % increase) accounted for the majority of the variation observed in consumption outcomes.

## Discussion

The principal conclusion of the modelling process is that interventions to increase F&V consumption in urban Kenya may not only provide benefits but also face challenges. Reducing perishability is unlikely to result in substantive increases in F&V consumption even accounting for uncertainty in parameter values, for two principal reasons. First, the assumed cost increase required to reduce product losses is larger than the cost savings from losses, so costs per kg – and thus product prices – increase. Under this scenario, there is no shift in the demand curve (as there would be from changes in preferences or income) for F&V, so a higher price reduces consumption. A second reason is that value chain actors (farmers, intermediaries and vendors) typically place orders with their suppliers to meet expected demand with an anticipated amount of product loss. Farmers are assumed to make planting decisions consistent with meeting expected orders from intermediaries but also accounting for expected product losses. Smaller post-harvest product losses therefore imply that less production is needed to meet expected demand from intermediaries, which can have the effect of increasing unit variable costs of production if there are economies of scale (lower costs per unit produced as production increases) as assumed for this analysis. The specific assumptions about behaviours and costs here could affect the simulated outcome of changes in perishability, so further and more specific assessments of such interventions are merited.

Increasing consumer awareness has modest impacts on F&V consumption at the mean assumed values of many parameters but would have more impact if the responsiveness of consumers to awareness is larger than the mean value assumed or if demand for F&V is less elastic (the percentage decrease in demand with a 1 % increase in price is lower). Efforts to increase consumer awareness are often promoted as a means to improve the quality of diets^(24)^, but evaluations of their effectiveness have shown mixed results^(5,25)^. The limited impact on F&V consumption from this intervention (an increase of less than 3 g/person/d) derives from the GMB workshop consensus that most Nairobi consumers are already aware of the relevant health benefits (so that only small increases in awareness are possible) and that the responsiveness of F&V consumption to increased awareness is relatively low. The percentage change in consumption associated with a 1 % change in awareness (‘elasticity’ value) is estimated as 0·6 based on stakeholder input.

Increasing farm yields appears to have the largest potential to increase F&V consumption, because the resulting lower farm prices increase demand by intermediaries and vendors. Prices initially fall throughout the supply chain but then increase for vendors and consumers as the effects on demand from emotional benefits and making good choices further enhance demand. Although this intervention is the most effective for increasing consumption, it also *markedly lowers farm profitability* compared to the status quo. This occurs because farms are assumed not to have much as much ability to modify their prices in response to changes in supply and demand as intermediaries or vendors, per the discussions in the second workshop. Large reductions in farmer profits could undermine attempts to increase consumption through implementation of yield increases.

Overall, our stochastic analyses suggest that substantive increases in F&V consumption are unlikely with reduced perishability under any assumed values for uncertain model parameters. However, there is a potential for substantively larger increases in F&V consumption than is predicted by the mean values of parameters for interventions to increase consumer awareness and farm yields. Moreover, there is overlap in the distributions of F&V consumption outcomes under uncertain parameter values for these latter two scenarios. Further work to define more narrowly the values of uncertain parameters would be beneficial to the identification of more effective interventions.

Much of the information necessary for the development of the quantitative model was not readily available from previous sources. Although stakeholders provided their assessments and uncertainty in this information was evaluated with the simulation model, the relatively large ranges of outcomes indicated by the stochastic analyses suggest that allocating resources to improved knowledge would be valuable. Three main areas merit further knowledge development. Additional information on the cost structures and prices through the value chain^(21)^ and their changes over time and in response to interventions would allow improved representation of core business performance metrics and likely behaviours. Information on the responsiveness of consumers to changes in factors such as quality, convenience and hygiene is very limited; extant studies often include ranking of importance of these factors but not a linkage to their impacts on purchases or consumption. Finally, the potential for change in each of the factors and associated costs would better inform scenario development and allow more refined use of the value chain linkages in the current simulation model.

The suggested next steps are to use the results of this modelling study to undertake a series of small exploratory studies to improve knowledge of value chain relationships, consumer purchasing and consumption behaviour and the potential for interventions to modify key value chain components and affect consumption behaviour. This information could be used to refine the analyses reported herein – to narrow the range of possible outcomes currently due to data uncertainty. Given the higher-level results of this study, complementary systems analytical approaches could be applied to assess implementation challenges^(28)^ and to establish protocols for monitoring, evaluation and learning^(28)^.

## Supplementary material

For supplementary material accompanying this paper, visit https://doi.org/10.1017/S1368980023002033
